# Supplemental effects of different production methods of pine needle additives on growth performance, intestinal environment, meat quality and serum of broiler chickens

**DOI:** 10.5713/ab.24.0042

**Published:** 2024-05-07

**Authors:** Yi-Qiang Chang, Seung-Kyu Moon, Yan-Qing Wang, Liu-Ming Xie, Hang-sul Cho, Soo-Ki Kim

**Affiliations:** 1 Department of Animal Science and Technology, Konkuk University, Seoul 05029, Korea; 2 State Key Laboratory of Food Science and Resources, Nanchang University, Nanchang 330047, China

**Keywords:** Broiler Chickens, Feed Additives, Growth Performance, Intestinal Flora, Pine Needle

## Abstract

**Objective:**

Pine needles are rich in many nutrients and exhibit antibacterial and antioxidant biological activities; however, the effects of different production methods of pine needle additives on the growth performance and intestinal flora of broiler chickens are not known.

**Methods:**

Normal diets were supplemented with pine needle fermentation juice (PNF), pine needle soaking juice (PNS), or pine needle powder (PNP), and the associated effects on growth performance, relative organ weights, intestinal development, intestinal histological morphology, intestinal flora, meat quality, and serum indicators in broiler chickens were observed.

**Results:**

The results showed that PNF, PNS, and PNP all significantly improved feed utilisation and promoted the growth and development of broilers. All three additives also significantly improved the structure of the intestinal flora, specifically increasing the diversity of bacteria; increasing the abundance of beneficial bacteria, such as *Faecalibacterium*, *Rikenella*, and *Blautia*; and decreasing the abundance of harmful bacteria, such as *Staphylococcus*. The antioxidant properties of pine needles were also found to intensify lipid metabolic reactions in the blood, thus leading to lower triglycerides and total cholesterol. Meanwhile, high doses of PNF reduced jejunum and ileum weights and also increased meat yellowness. Lastly, none of PNF, PNS, or PNP had an effect on relative organ weights or intestinal histological morphology.

**Conclusion:**

The addition of pine needles to the diet of broiler chickens can effectively promote their growth performance as well as improve their intestinal flora and serum status without side effects; in particular, the dose of 0.2% of either PNF and PNS is expected to have the capacity to replace growth-promoting antibiotics as diet additives.

## INTRODUCTION

In recent years, the misuse of antibiotics has jeopardised public health and safety. Antibiotics are mainly used in agriculture as growth promoters and disease preventive agents in animals, and such use has been banned in some countries of the European Union (EU), as this usage leads to increased drug resistance over time that gradually weakens the efficacy of the drugs, while also leading to an increased risk of transmission of zoonotic diseases due to increased antimicrobial resistance in humans as a result of downstream exposure [1]. The current alternatives to antibiotics for such use in farm animals mainly comprise antimicrobial vaccines, immunomodulators, phages and their lysosomes, antimicrobial peptides, inhibitors of targeted pathogenicity (bacterial population sensing, biofilms, and virulence), and feed enzymes. However, all of these treatments can have side effects, including intestinal damage and inhibition of beneficial bacteria in the intestine. Therefore, there is an urgent need for the development of safe, low-toxicity, and effective alternatives to antibiotics that are derived from natural substances [2].

Plants contain a variety of natural components, and their varying natural compositions can contribute to increased biodiversity and thus help control the growth of microorganisms such as bacteria and molds [3]. They are even more widely used in the field of animal feed and additives. Essential oils can further promote the production of digestive secretions and nutrient absorption in the intestinal tract of swine and poultry, thus reducing pathogenic stress in the intestine [4]. Feeding a mixture of barley and wheat extracts has been shown to increase lipase activity in older broiler chickens by 38% to 46% on a basal basis [5]. Another study showed that the addition of extracts from essential oils of sage, thyme, cinnamon and cayenne promote feed digestibility among broilers without any toxicological effects [6].

Pine needles are rich in nutrients such as proteins, minerals, and vitamins, and they possess anti-inflammatory [7], antioxidant, anti-mutagenic, and anti-tumor [8], and antimicrobial properties [9]; these properties have led to their widespread use as feed additives. For example, the feed addition of pine needle extract as a supplement has been shown to significantly improve the intestinal flora of chickens [10]. The addition of low doses of pine needle extract has also been shown to lead to heavier yolk weights in laying hens [11].

Fermentation is a commonly used method to increase the shelf life of food and improve its flavor, and the bacteria involved in fermentation activities produce bioactive peptides that remove non-nutrients and provide health benefits [12]. Molasses is rich in carbohydrates and fats, minerals, and other substances; can be used as silage; and can be added to fermented plants to increase the content of internal lactic acid bacteria and reduce the content of organic acids and ammonia [13]. Food-based plant powders are often used in food manufacturing, as powder processing minimises nutrient loss and facilitates handling for easier absorption by the body [14]. Many of the previous studies have focused primarily on the effects of pine needle powder and pine needle extracts on the growth performance of broilers, while fewer studies have examined or compared the effects of different types of pine needle additives on broilers. Therefore, the present experiment investigated the effects of three pine needle feed additives made using different production methods. In particular, fermented pine needle (pine needle ferment, PNF), soaked pine needle (pine needle soak, PNS), and powdered pine needle (pine needle powder, PNP) were analysed in terms of their effects on growth performance, relative organ weights, intestinal development, intestinal tissue morphology, intestinal microflora, meat quality, and serum of broilers. Adverse reactions and their side effects were also monitored.

## MATERIALS AND METHODS

The current study was conducted in accordance with the ARRIVE (https://arriveguidelines.org) guideline report.

### Ethical statement

All animal care procedures were approved by the Institutional Animal Care and Use Committee of Konkuk University (Accreditation number: KUB23004). The experiment was conducted on an individual broiler farm in Chungju, South Korea, which is where all rearing conditions were carried out according to the experimental guidelines, and the appropriate breeding licence was obtained.

### Experimental design and husbandry

To begin, 630 one-day-old male Ross 308 broiler chicks (with an initial weight of 45 g±0.5) were reared continuously for 5 weeks. The chicks were divided into seven groups: i) basal diet (negative control; NC); ii) basal diet + 0.02% Enradin antibiotic products containing enramycin provided by Woogene B&G (Seoul, Korea) (positive control; PC); iii) basal diet + 0.2% pine needle fermentation juice (PNF) (T1); iv) basal diet + 0.4% PNF (T2); v) basal diet + 0.2% pine needle soaking juice (PNS) (T3); vi) basal diet + 0.4% PNS (T4); and vii) basal diet + 0.3% pine needle powder (PNP) (T5). Each treatment group had six replicates, where each replicate group housed 15 chicks with similar initial body weights (BW). The provision of basal rations was divided into two phases, pre-feeding and post-feeding, while the composition and calculated chemical composition of corn and soybean meal-based basal rations were formulated according to the 2017 Korean Poultry Feeding Standards and are listed in Table 1 [15].

All chicks were housed on a concrete floor covered with 5 cm of bran; they had access to an unlimited supply of food and water throughout the feeding cycle, as described in the previous feeding program. The relative humidity was set at 60% to 65%, and 24 hours of continuous light was provided. The temperature of the chicken house was initially controlled at 33°C before being decreased by two degrees per week until the end of the feeding period.

### Preparations of feed additives

Three kinds of feed additives of pine needle were prepared by Celltec Co. Ltd. (Chungju, South Korea).

i) Pine needle fermentation juice: 700 g of PNS and 350 g of molasses were mixed with water to 5 L. *Lactobacillus plantarum* (*L. plantarum*) SK4315 and *Saccharomyces cerevisiae* (*S. cerevisiae*) SK3587 (6 to 7 logCFU/mL) were then inoculated and fermented as solid state for 72 hours at 30°C. The prepared solution was then mixed 1:1 with soybean meal and dried in a drying oven at 50°C to obtain the finished product.
ii) Pine needle soaking juice: Pine needle soaking juice was produced by Pinebio Co. Ltd. (Bongwha, Korea). The juice was soaked by sugar at a ratio of 1:1 without the addition of water for one year at room temperature. Pine needle juice of 0.7 L and water of 4.3 L were mixed, after which soybean meal was added and mixed to 10 kg to obtain the finished product.
iii) Pine needle powder: Natural fine needles were chopped and dried with a dryer for 1 day at 50°C. Next, they were directly ground into powder with a blender. Then, 700 g of the pine needle powder and 9.3 kg of soybean meal were mixed to 10 kg to obtain the finished product.

### Growth performance

All broilers were weighed weekly by group and recorded separately. Records were kept of the body weight (BW) of the broilers in each treatment group during the three rearing stages: (0 to 7 day), (0 to 21 day), and (0 to 35 day). Body weight gain (BWG) was measured as the change in the weight of the pullets from the time they were housed to the time they were released. Average daily gain (ADG) is the BWG divided by the number of days. Feed intake (FI) is the weight of the remaining ration for the day minus the weight of the feed offered on the previous day. Feed conversion ratio (FCR) is FI divided by ADG. All indicators were measured on the premise that the number of deaths was recorded on a daily basis.

### Organ sampling and weight

At the end of rearing, one broiler per replicate that had a weight close to the average weight of the group was selected for sample collection. Specific samples collected included liver, spleen, Fabricius bursa, breast, leg (single right side), small intestine (duodenum, jejunum, and ileum) and cecum, and blood. Blood collection was performed first; anticoagulated blood collection tubes were prepared and labeled sequentially before blood collection. Immediately after death by carbon dioxide asphyxiation, blood was collected by cardiac puncture, and 10 mL of fresh blood was drawn into BD Vacutainer K2E (ethylenediaminetetraacetic acid) blood tubes and immediately stored at 4°C for preservation. Using a serum centrifuge, the serum was separated by centrifugation at 1,500 rpm for 10 minutes (HA-1000-3; Hanil Science Medical, Daejeon, Korea). Breast, thigh (single right side), liver, spleen, bursa, and small intestine and cecum samples were all stored at 4°C immediately after dissection.

The collected livers, spleens, Fabricius bursa, breasts and whole leg meat (right side) were weighed as a whole after having excess fat removed using an electronic balance (EL4002; Mettler Toledo, Zürich, Switzerland). Results were expressed as the weight ratio of chicken meat per 100 g of live BW.

### Internal morphology of intestine

The small intestine and cecum are used to analyse intestinal characteristics. The small intestine is divided into three sections: i) duodenum: from the gizzard to the beginning of the mesentery; ii) jejunum: from the farthest insertion point of the mesentery to 5 cm before the Meckel's diverticulum; and iii) ileum: from 5 cm after the Meckel's diverticulum to the ileocecal junction. Samples of the small intestine and cecum were collected and measured in terms of weight and length. The results were expressed as the weight ratio of chicken meat per 100 g of live BW.

After fasting for 12 hours prior to dissection, intact jejunum and ileum midsections of 5 cm in length were collected with three replicates for each treatment group. The center portion of the jejunum and ileum was then cut at about 3 cm, and the intestinal surface and contents were rinsed with phosphate buffer solution of pH 7.2. The samples were immersed in a 1% formalin solution, then in a 5% formalin solution, and finally in a 10% formalin solution. Next, the processed samples were placed in Falcon sample tubes and stored in ice at 4°C before being transported to the laboratory. The next sample processing step was sectioning, and the sections were stained with standard hematoxylin-eosin (H&E) stain. Villus height (VH) and crypt depth (CD) were determined using a microscope (BX43; Olympus, Tokyo, Japan) and corresponding software (DIXI Science; Daejeon, Korea). VH is measured as the depth from the tip of the villus to the villus-crypt junction, while CD is measured from that junction to the base of the crypt, and VH:CD is the ratio of the two, both of which are expressed in μm. In total, five section lengths were measured, and the average value was taken.

### Intestinal microbiota

Polymerase chain reaction conditions, DNA extraction, bioinformatics, and next generation sequencing sequencing analysis were carried out following a previously described method [16]. In short, a PowerSoil DNA isolation kit (Mobio Laboratories, Inc., Carlsbad, CA, USA) was first used for the isolation of genomic DNA. Next, the V3–V4 region of the bacterial 16S rRNA gene was amplified by 341F and 785R primers. Sequencing was then performed using the Illumina Miseq platform through the commercial service of Macrogen (Seoul, Korea). To compare alpha diversity, operational taxonomic units, Chao1, Shannon, and Gini-Simpson indexes were checked. For beta diversity analysis, principal coordinate analysis (PCoA) and weighted UniFrac distance matrix-based unweighted pair-group mean average (UPGMA) analysis were used.

### Analysis of meat quality

To determine the quality of chicken meat, the collected samples of chicken breasts were analysed. The pH value was measured by inserting the measuring needle of a pH meter (Hanna Instruments, Nusfalau, Romania) to a depth of 1 cm inside the chicken breasts and calculating the value from the displayed value; this was measured three times for each sample, recorded sequentially. Cooking loss: samples were placed in polyethylene bags and heated in a water bath (C-WBE; Chang Shin Co., Seoul, Korea) at 75 degrees Celsius for 30 minutes, then left to cool at room temperature for 10 minutes; the difference in weights before and after the heating was compared to calculate the amount of loss.


$$\begin{array}{l} {}*\;\text{Cooking loss }(\%)\\ =(\text{Sample weight before cooking}\\ -\text{Sample weight after cooking})\\ /(\text{Sample weight before cooking})\times 100 \end{array}$$

Regarding the display of the colorimetric values of the meat, a colorimeter (Chromameter, CR210, Minolta, Japan) was used to measure the surface of the samples, and the L* value for lightness, a* value for redness, and b* value for yellowness were used. For standard color, a chromaticity calibration plate was used that had an L* value of 97.69, an a* value of −0.43, and a b* value of +1.98.

### Blood profiles

The chemical compositions of the blood samples taken from broilers in this experiment were analysed using an automated dry chemical analyser for veterinary use (CHEM7000i, Tokyo, Japan) at the Biological Center of Konkuk University Research Facility (Seoul, Korea). This analysis measured gamma-glutamyl transpeptidase, glutamic oxaloacetic transaminase, glutamic pyruvate transaminase, glucose, blood urea nitrogen, creatine, triglycerides (TG), total protein, albumin, uric acid), total cholesterol (TCHO), and high-density lipoprotein (HDLC).

### Statistical analysis

Data were examined as a completely randomised design using SAS 9.4's PROC mixed process (SAS Institute, Cary, NC, USA). Growth performance parameters are used as experimental units for each pen of chickens. The results are expressed as mean and standard error of mean (SEM). Statistical significance is considered to be extremely significant if p<0.01, significant if p<0.05, and to have a trend towards statistical significance if 0.05<p<0.1. ^a–d^ Mean within a column within a main effect are significantly different.

## RESULTS

### Growth performance

Table 2 shows the effects of the addition of PNF, PNS, and PNP on growth performance in broiler chickens. As can be seen in the Table, at 0–35 days, the BW of all treatment groups were higher than that of the NC group (p<0.05). BWG and ADG were also higher in the treatment groups than they were in the NC group at mid to late rearing, and this was shown to be a dose-dependent relationship (p<0.05). On the other hand, the FI values of the T3 and T5 treatment groups were slightly lower than the NC group at the beginning of the feeding period, but the changes in FI were positively correlated with the results of the FCR. At the middle and end of the feeding period, both FI and FCR of the treatment groups were lower than those of the NC group (p<0.05). The addition of PNP showed most low tendency of FCR compared to other treatments with no significance.

### Relative organ weights

**Figure 1:**
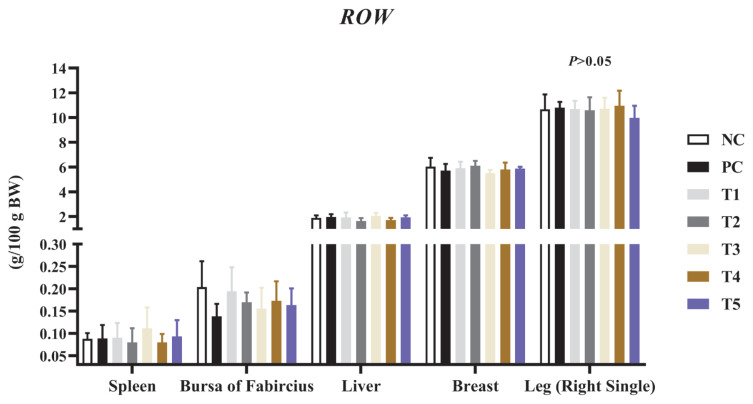
Effect of the addition of PNF, PNS, and PNP on relative organ weights. NC, negative control; PC, positive control; T1, 0.2% pine needle fermentation juice (PNF); T2, 0.4% PNF; T3, 0.2% pine needle soaking juice (PNS); T4, 0.4% PNS; T5, 0.3% pine needle powder (PNP). ROW, ratio of organ weights.

Figure 1 shows the effect of the addition of PNF, PNS, and PNP on the relative organ weights of broiler chickens. None of the treatment groups containing showed any significant difference (p>0.05) in any of the following five analysed indexes.

### Intestinal development

**Figure 2:**
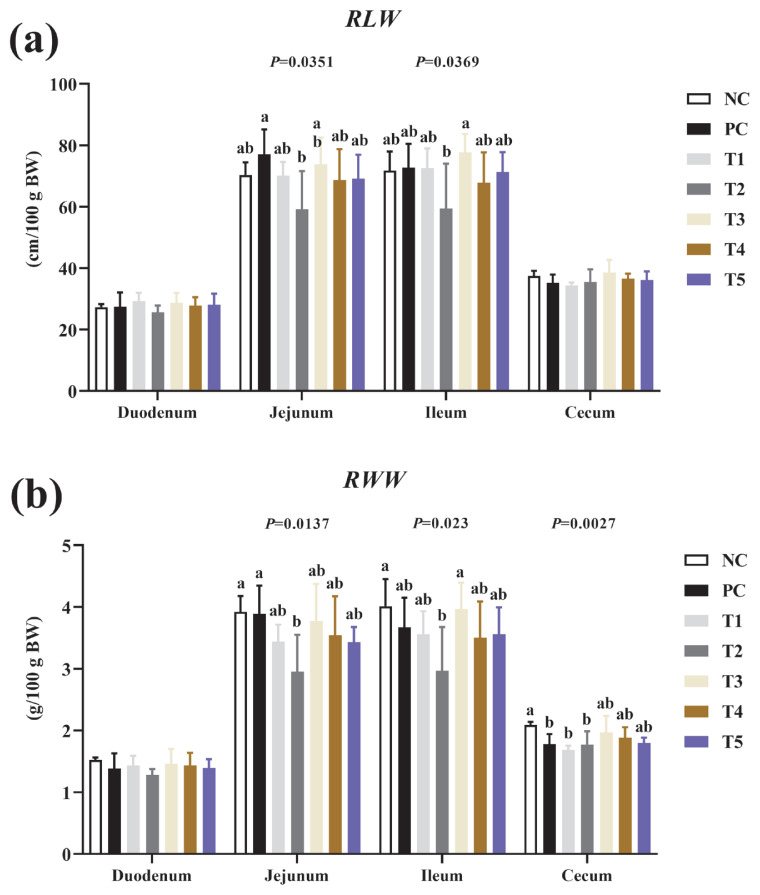
Effects of the addition of PNF, PNS, and PNP on intestinal development. NC, negative control; PC, positive control; T1, 0.2% pine needle fermentation juice (PNF); T2, 0.4% PNF; T3, 0.2% pine needle soaking juice (PNS); T4, 0.4% PNS; T5, 0.3% pine needle powder (PNP). (a) RLW, ratio of intestinal length to body weight; (b) RWW, ratio of intestinal weight to body weight. ^a,b^ Means with different letters are significantly different (p<0.05).

Figure 2 shows the effects of the addition of PNF, PNS, and PNP on intestinal development in broiler chickens. PNF, PNS, and PNP had no significant effect on ileum or jejunum lengths (p>0.05). Meanwhile, PNS and PNP treatments had no significant effect on the relative weights of jejunum, ileum, or cecum (p>0.05). However, a high dose of PNF treatment significantly decreased the relative weights of jejunum and ileum (p<0.05).

### Histotypic morphology of jejunum and ileum

**Figure 3:**
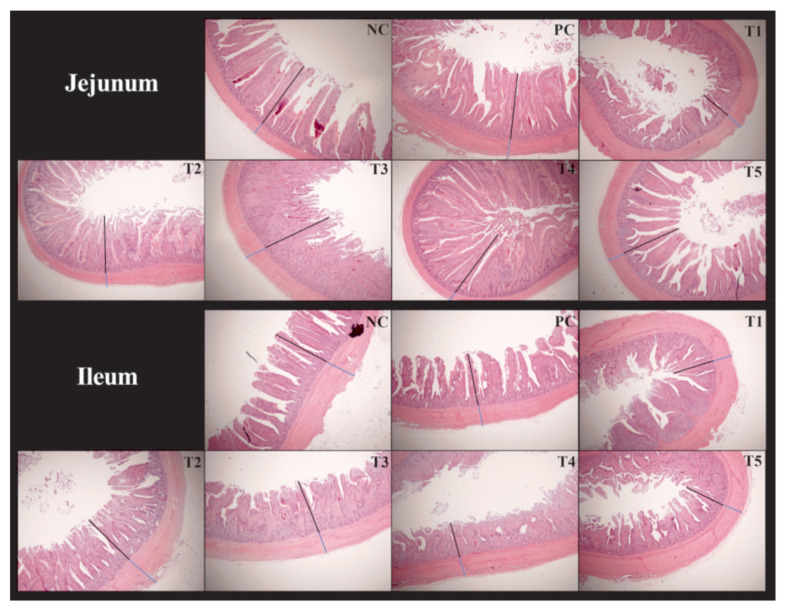
Histological morphology of the intestine viewed under a microscope. NC, negative control; PC, positive control; T1, 0.2% pine needle fermentation juice (PNF); T2, 0.4% PNF; T3,0.2% pine needle soaking juice (PNS); T4, 0.4% PNS; T5, 0.3% pine needle powder (PNP). Black: villus height; Blue: crypt depth. Observations were made using a microscope ×40. Original scale bar = 200 μm.

**Figure 4:**
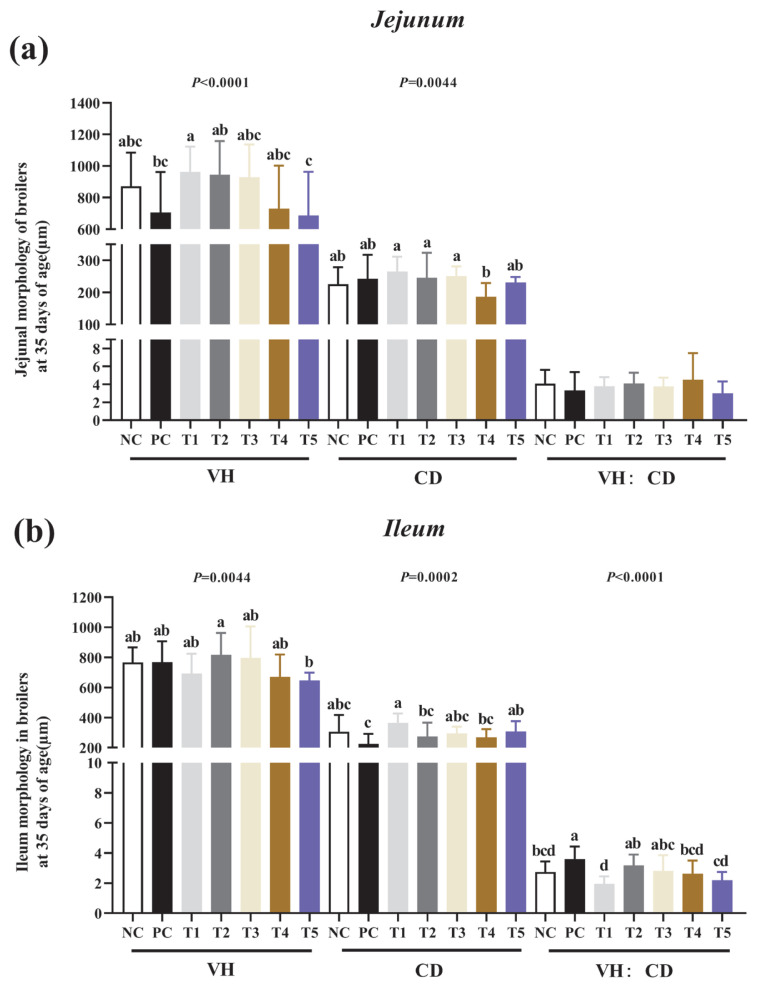
Effect of the addition of PNF, PNS, and PNP on intestinal histological morphology. NC, negative control; PC, positive control; T1, 0.2% pine needle fermentation juice (PNF); T2, 0.4% PNF; T3, 0.2% pine needle soaking juice (PNS); T4, 0.4% PNS; T5, 0.3% pine needle powder (PNP). (a) Jejunum intestinal histomorphology; (b) Ileum intestinal histomorphology. VH, villus height; CD, crypt depth; VH:CD, villus height: crypt depth. ^a–d^ Means with different letters are significantly different (p<0.05).

Figures 3 and 4 show the effect of the addition of PNF, PNS, and PNP on the intestinal histological morphology of broiler chickens. The length of villi and depth of crypts in the jejunum and ileum of broilers was unchanged among PNF, PNS, and PNP (p>0.05).

### Intestinal microflora

**Figure 5:**
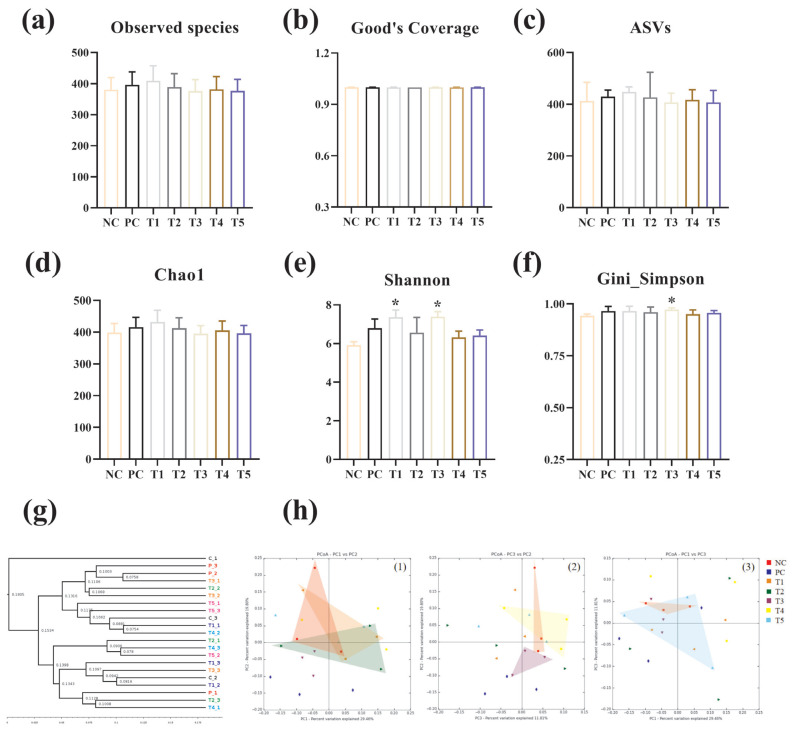
Effect of the addition of PNF, PNS, and PNP on the diversity of intestinal flora. NC, negative control; PC, positive control; T1, 0.2% pine needle fermentation juice (PNF); T2, 0.4% PNF; T3, 0.2% pine needle soaking juice (PNS); T4, 0.4% PNS; T5, 0.3% pine needle powder (PNP). (a–f) Alpha diversity index of cecum microorganisms. (g and h) Phylogenetic tree of the cecum microbiota and principal coordinate analysis based on Bray-Curtis distance, where the confidence interval is 95%. * p<0.05.

Figure 5 shows the alpha-diversity indices (Observed species, Good's Coverage, ASVs, Chao1, Shannon, and Gini-Simpson) for the gut microbiota of broilers ingesting PNF, PNS, and PNP. In the T3 group, the Shannon and Simpson indices were significantly stronger than in the NC group, while in the T1 group, the Shannon index is stronger than in the NC group (p<0.05). Regarding beta diversity (Figures 5g and 5h), the PCoA plots with weighted Unifrac distances clearly showed that the microbial colonies formed confidence zones between groups, and the confidence triangles of the bacterial communities in the T3 treatment group area deviated significantly from those of the NC group. Meanwhile, PD_whole_tree (UPGMA) showed comparable species homology between the other treatment groups and the NC group.

**Figure 6:**
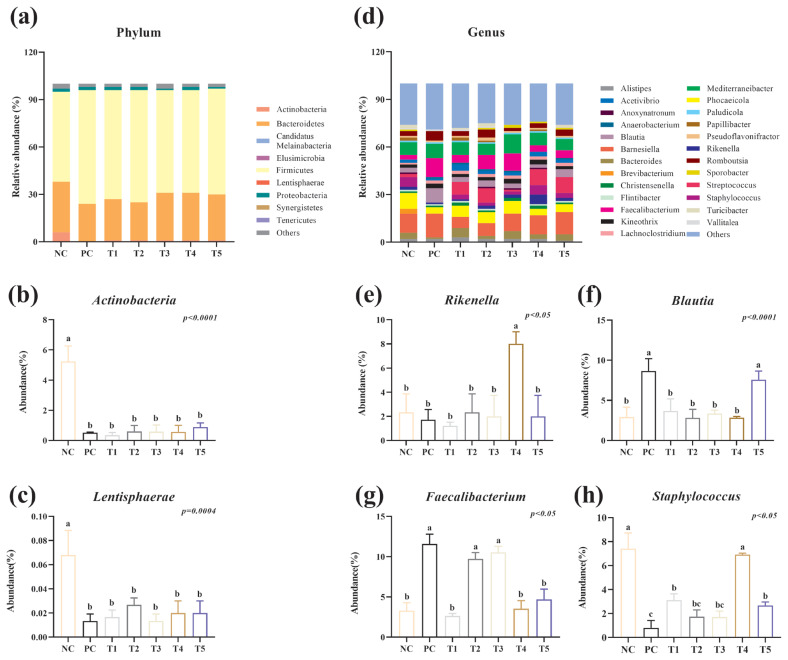
Effect of the addition of PNF, PNS, and PNP on the abundance of intestinal flora at the phylum and genus levels. NC, negative control; PC, positive control; T1, 0.2% pine needle fermentation juice (PNF); T2, 0.4% PNF; T3, 0.2% pine needle soaking juice (PNS); T4, 0.4% PNS; T5, 0.3% pine needle powder (PNP). (a–c) Relative abundances of the gut microbiota at the phylum level. (d–h) Relative abundances of the gut microbiota at the genus level. ^a–c^ Means with different letters are significantly different (p<0.05; n = 3).

To determine the changes in the gut flora after PNF, PNS, and PNP interventions, the microbiological components were analysed. The microflora in the cecum of broiler chickens at the phylum level mainly consisted of *Bacteroidetes* (66.91%) and *Firmicutes* (27.91%). Two superior species, *Firmicutes* and *Bacteroidetes*, accounted for ~90% of the total microorganisms in relative abundance. Meanwhile, PNF, PNS, and PNP may reduce the abundance of some *Actinobacteria* and *Lentisphaerae*, which contrasts with the findings of the NC group (p<0.05) (Figures 6a–6c).

Figure 6d depicts the classification components of the gut flora at the genus level. *Bacteroides* (11.24%) and *Mediterraneibacter* (8.29%) stood out from the rest of the microflora, and these two bacteria accounted for the largest percentage. Four dominant bacterial families were chosen to analyse the variations in the gut microbiota compositions in various samples (Figures 6e–6h). The relative abundance of the beneficial bacteria, *Rikenella* in group T4, *Blautia* in group T5, and *Faecalibacterium* in groups T2 and T3, was significantly increased compared to the corresponding abundance in the NC group. As for harmful bacteria *Staphylococcus*, it was significantly lower in all but the T4 group compared to the NC group.

### Meat quality

Table 3 presents the effect of the addition of PNF, PNS, and PNP on the meat quality of broiler chickens. Compared to the NC group, none of the PNS or PNP interventions had significant effects on meat quality indexes in broilers (p>0.05), but the high dose of PNF increased the yellowness of the meat (p<0.05).

### Serum indicators

**Figure 7:**
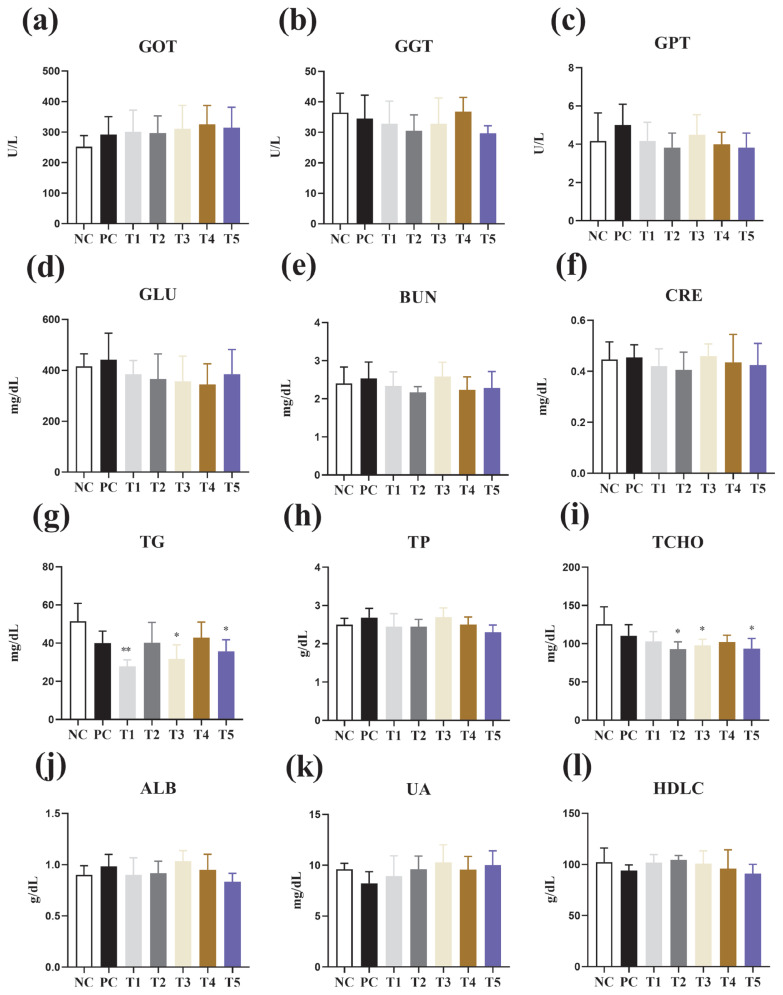
Effect of the addition of PNF, PNS, and PNP on serum. NC, negative control; PC, positive control; T1, 0.2% pine needle fermentation juice (PNF); T2, 0.4% PNF; T3, 0.2% pine needle soaking juice (PNS); T4, 0.4% PNS; T5, 0.3% pine needle powder (PNP). (a) Glutamate oxaloacetate transaminase (GOT). (b) Gamma-glutamyl transpeptidase (GGT). (c) Glutamate pyruvate transaminase (GPT). (d) Glucose (GLU). (e) Blood urea nitrogen (BUN). (f) Creatine (CRE). (g) Triglycerides (TG). (h) Total protein (TP). (i) Total cholesterol (TCHO). (j) Albumin (ALB). (k) Uric acid (UA), (l) High density lipoprotein (HDLC). * p<0.05. ** p<0.01.

Figure 7 shows the effect of feed additions of PNF, PNS, and PNP on serum indices of broiler chickens. Low doses of PNF, PNS, and 0.3% PNP reduced TG levels compared to the NC group, while high doses of PNF and low doses of PNS and PNP reduced TCHO levels (p<0.05).

## DISCUSSION

The results of the study showed that PNF, PNS, and PNP all promoted growth performance in broilers, with PNF having the best effects, while PNP and PNS also had desirable effects and can thus be recommended as well. The fermentation of the pine needle mixture using *L. plantarum* and *S. cerevisiae* in this experiment has previously been shown to have good antimicrobial activity and antioxidant activity, part of the reason for this is that mixed fermentation of *L. plantarum* and *S. cerevisiae* better reduces ANFs in pine needles and increases the concentration of probiotics, enzymes and metabolites [17]. Moreover, using *Bacillus subtilis* with antimicrobial and antioxidant properties has been shown to improve the expression of antioxidant enzyme genes in broiler chickens and significantly improve their growth performance [18]. The addition red pine bark extracts improved the growth performance of broilers, and the higher dose showed better outcomes than the lower dose [19]. Pine needles contain essential oils, terpenes, and polyphenols, which promote the absorption and utilization of nutrients in the digestive tract, and the unique aroma of pine needles improves the flavour of the feed and enhances appetite [20]. Previous studies have reported that proanthocyanidin and shikimic acid in pine needles can control and regulate the expression of cellular pro-inflammatory factors and improve the body's immune response [21,22].

In the present work, PNF, PNS, and PNP did not show any differences in liver, spleen, and bursa. The liver is the largest paraglandular gland in the avian digestive system, and it plays an important role in lipid synthesis as well as in the storage and conversion of metabolites [23]. Pine needle extract has mitogenic activity, which promotes the expression of PCNA (proliferating cell nuclear antigen) and Ki-67 proteins, thus resulting in hepatocyte proliferation, in addition to the anti-oxidative stress properties of pine needles, which have the potential to influence and promote liver regeneration [24]. Spleen weight is associated with proliferation and apoptosis of immune cells, and an immune response is triggered when a large amount of antigen enters; Pine needles also have immune-boosting properties, however, it is tentatively unclear whether the reduction in the weight of the spleen in mice is related to the pine needle distillate [25]. However, red pine bark has been shown to increase the weight of the bursa and spleen, modulate the immune response, and inhibit pathogen proliferation, as well as reduce host tissue damage [19]. The carcass weights in this study did not change, which is consistent with previous findings [26].

The small intestine stage absorbs most of the nutrients that enter the body, and the degree of intestinal development affects the efficiency of nutrient absorption during the growth and development stages of the organism [27]. The results indicated that the interventions of PNS and PNP had no effect on intestinal growth and development, while high doses of PNF resulted in lower intestinal weights. Pine needles contain cellulose and lignin compounds, the combination of which affects the absorptive capacity of the intestines; this is relevant in birds, because they regulate the length and weight of their gastrointestinal tract by judging the fiber content of their food [28].

Previous research has shown that VH and CD are related to the digestive capacity of the intestine, elevated villus height increases the area of intestinal nutrient absorption, and increased VH:CD ratio improves intestinal morphology, thus contributing to stress resistance and intestinal barrier function in animals [29]. The results indicate that PNF, PNS, and PNP did not affect the histological morphology of the intestine. In conjunction with the results of previous studies, these findings indicate that, although feeding fibrous diets improves the internal environment of the intestine, it has little effect on the histological morphology of the intestine [30].

The intestinal microbiota profoundly influences intestinal homeostasis, not only affecting intestinal metabolites but also regulating intestinal immune homeostasis [31]. The study of alpha and beta indices were analysed herein, with the results showing that PNF, PNS, and PNP increased the homology and diversity of microorganisms in the cecum, with low doses of PNS being the most effective. At the phylum level, *Bacteroidetes* and *Firmicutes* were above 90%. Afterwards, PNF, PNS, and PNP reduced the abundance of *Actinobacteria* and *Lentisphaerae*. *Mycobacterium tuberculosis* of the genus *Actinobacteria* is a strong pathogen that can cause infection in chickens [32]. *Lentisphaerae* are predominantly found in marine environments, but they also survive in feces and are often difficult to isolate in culture, so their main physiological and ecological properties are tentatively unknown [33]. Moreover, 0.4% PNS significantly increased the abundance of *Rikenella*. Some reports suggest that *Rikenella* may be a target or biomarker for certain diseases, such as diabetes and obesity, and that the core function is to control butyric acid synthesis in the body and to promote metabolism; prior studies have also shown that the addition of a pine needle additive to cattle feed transiently increased the amount of *Rikenella* in the rumen microflora [34]. Herein, PNP increased the abundance of *Blautia*, high doses of PNF and low doses of PNS increased the abundance of *Faecalibacterium*, and all pine needle additives except for high doses of PNS decreased the abundance of *Staphylococcus*. *Rikenella*, *Blautia* and *Faecalibacterium* are typically recognised as beneficial bacteria [35,36]. Pine needles are able to inhibit the growth of *Staphylococcus*, and studies have shown that the mangiferic acid contained in pine needles inhibits the growth of *Staphylococcus aureus* [37]. Therefore, it is suggested that the intervention of PNF, PNS, and PNP changes the structure of the intestinal flora, increases its diversity, promotes the growth of beneficial bacteria in the intestinal tract, and inhibits the growth of harmful bacteria. In particular, PNS showed more effective microflora.

Altogether, this study showed that supplementation with PNF, PNS, and PNP led to reduced oxidative damage. a* stands for redness and b* for meat colour, which is yellow, whereas high and low values of a* and b* describe the process of the deterioration of meat colour over time and reflect the concentration of myoglobin in the meat and their redox status [38]. Myoglobin degradation is accelerated with increasing temperature and time; the concentration of pine needles also affects redox reactions [38]. However, curcumin in pine needles causes pigmentation and an increase in yellowness values [39]. This explains the increase in the yellowness value in this study.

The determination of blood composition through laboratory testing is an important procedure that assists in the diagnosis of various poultry diseases and disorders. Cholesterol and triglycerides are important markers of lipid metabolism [20]. Moreover, there are certain phenolic compounds present in pine needles, such as terpenoid, which hinders fat and cholesterol biosynthesis, enhances *in vivo* utilisation by inhibiting lipids and oxidation, inhibits fat absorption and increases fat excretion through micelle formation and blockage in the small intestine, and plays an inhibitory role in the reabsorption of bile acids, thus affecting lipid metabolism *in vivo* [29]. Pine needles cause a reduction of synthetic enzymes in the blood, inhibit oxidative free radicals, and enhance lipid peroxidation, ultimately resulting in lower TG and TCHO levels [40]. PNF, PNS, and PNP all improved serum status in broilers, which is consistent with previous findings [40].

In conclusion, the results showed that PNF, PNS, and PNP enhanced the dietary utilisation of broilers while increasing growth performance with improved gut flora and serum status, with effects comparable to the effect of antibiotic treatment. Among the tested additives, the addition of PNF and PNS with 0.2% content was more prominent. More importantly, PNF, PNS, and PNP have no toxic side effects, so they can safely be used as natural feed growth promoters.

## Figures and Tables

**Table 1:** Ingredients and compositions of the experimental diets

Item	Starter (1 to 21 d)	Grower (21 to 35 d)
Ingredient (%)		
Corn	50.75	53.511
Wheat	5.00	5.000
SBM (IMP)	34.09	31.331
Tallow	4.99	6.117
L-methionine, 98%	0.32	0.247
Lysine-Syn, 24%	0.96	0.244
L-threonine, 98%	0.13	0.027
Limestone	1.59	1.535
MDCP	1.29	1.156
Choline Cl, 50%	0.10	0.071
Salt	0.28	0.280
Vitamin premix^1)^	0.15	0.150
Mineral premix^2)^	0.15	0.150
Phytase	0.02	0.020
NaHCO3	0.16	0.161
Chemical composition calculated		
CP (%)	21.00	19.00
Crude fiber (%)	2.88	2.96
Ca (%)	0.90	0.85
Available phosphorus (%)	0.35	0.32
Total Lys (%)	1.37	1.10
Total TSAA (%)	0.99	0.87
Total Thr (%)	0.90	0.74
AMEn (kcal/kg)	3,050	3,146

SBM, soybean meal; IMP, inosine monophosphate; MDCP, monodicalcium phosphate; CP, crude protein; TSAA, total sulfur amino acids; AMEn, apparent metabolic energy.

1) Vitamin premix provides the following (per kg of diet): Vitamin A, 5,500 IU; Vitamin D_3_, 1,100 IU; vitamin E, 10 IU; riboflavin, 4.4 mg; vitamin B_12_, 12 mg; nicotinic acid, 44 mg; menadione, 1.1 mg; biotin, 0.11mg; thiamine, 2.2 mg; ethoxyquin, 125 mg.

2) Mineral premix provides the following (per kg of diet): Mn, 120 mg; Zn, 100 mg; Fe, 60 mg; Cu, 10 mg; Se, 0.17 mg; I, 0.46 mg; Ca, min: 150 mg, max: 180 mg.

**Table 2:** Effect of the addition of PNF, PNS, and PNP on growth performance

Item	Treatment^1)^							SEM^2)^	p-value

	NC	PC	T1	T2	T3	T4	T5		
BW (g/bird)									
Day 7 (initial weight)	155.95	154.56	156.99	157.79	158.33	160.97	161.08	3.00	0.683
Day 21	820.07	861.36	864.99	842.89	848.15	853.03	873.64	13.30	0.1415
Day 35	1,786.03^b^	1,961.5^a^	2,033.69^a^	1,981.55^a^	1,930.59^ab^	1,950.72^a^	1,945.6^a^	35.01	0.0012
BWG (g/bird)									
Grower phase	780.73	822.04	825.65	803.55	808.82	813.71	834.29	13.30	0.1414
Finisher phase	965.96^b^	1,100.13^ab^	1,166.71^a^	1,138.66^a^	1,082.45^ab^	1,097.69^ab^	1,071.96^ab^	32.29	0.0043
Total phase	1,746.68^b^	1,922.17^a^	1,994.36^a^	1,942.21^a^	1,891.26^ab^	1,911.4^a^	1,906.25^a^	35.02	0.0012
ADG (g/d/bird)									
Grower phase	37.18	39.14	39.32	38.27	38.51	38.75	39.73	0.63	0.1413
Finisher phase	69.0^b^	78.58^ab^	83.48^a^	81.34^a^	77.32^ab^	78.41^ab^	76.57^ab^	2.31	0.0043
Total phase	49.9^b^	54.92^a^	56.98^a^	55.49^a^	54.03^ab^	54.61^a^	54.47^a^	35.02	0.0012
FI (g/d/bird)									
Grower phase	69.01^a^	50.26^b^	62.43^ab^	61.78^ab^	57.83^b^	60.72^ab^	56.22^b^	2.09	0.0014
Finisher phase	197.06^a^	144.23^b^	150.42^b^	149.34^b^	147.39^b^	146.87^b^	142.57^b^	2.56	<0.0001
Total phase	120.23^a^	91.45^b^	97.63^b^	96.8^b^	93.66^b^	95.18^b^	90.76^b^	1.56	<0.0001
FCR (each bird)									
Grower phase	1.87	1.44	1.59	1.62	1.5	1.57	1.42	0.06	<0.0001
Finisher phase	2.87^a^	1.84^b^	1.8^b^	1.85^b^	1.91^b^	1.88^b^	1.88^b^	0.07	<0.0001
Total phase	2.41^a^	1.67^b^	1.72^b^	1.75^b^	1.74^b^	1.75^b^	1.67^b^	0.05	<0.0001

PNF, pine needle fermentation juice; PNS, pine needle soaking juice; PNP, pine needle powder; BW, body weight; BWG, body weight gain; ADG, average daily gain; FI, feed intake; FCR, feed conversion ratio.

1) NC, negative control; PC, positive control; T1, 0.2% PNF; T2, 0.4% PNF; T3, 0.2% PNS; T4, 0.4% PNS; T5, 0.3% PNP.

2) SEM, standard error of mean.

a,b Mean within a row within a main effect are significantly different (p<0.05).

**Table 3:** Effect of PNF, PNS, and PNP on meat quality

Item	Treatment^1)^							SEM^2)^	p-value

	NC	PC	T1	T2	T3	T4	T5		
pH	5.79	5.83	5.75	5.67	5.77	5.81	5.76	0.06	0.614
Cooking loss	15.85	16.31	19.80	19.97	18.37	19.36	19.12	1.43	0.2538
L*	61.39	60.43	61.57	62.45	60.88	60.41	61.52	0.53	0.0973
a*	1.91^abc^	1.56^abc^	1.34^c^	2.17^ab^	1.54^bc^	2.3^a^	1.37^c^	0.18	0.0002
b*	2.7^b^	2.56^b^	3.32^ab^	3.81^a^	3.06^ab^	2.87^b^	3.26^ab^	0.21	0.0008

PNF, pine needle fermentation juice; PNS, pine needle soaking juice; PNP, pine needle powder; L*, lightness; a*, red greenness; b*, yellow blueness.

1) NC, negative control; PC, positive control; T1, 0.2% PNF; T2, 0.4% PNF; T3, 0.2% PNS; T4, 0.4% PNS; T5, 0.3% PNP.

2) SEM, standard error of mean.

a–c Mean within a row within a main effect are significantly different (p<0.05).

## Data Availability

The datasets analyzed during the current study are available in the NCBI (National Center for Biotechnology Information) repository (https://www.ncbi.nlm.nih.gov/). Submission Numbers No. SUB13913931, Accession Numbers No. PRJNA 1029779. The data analyzed during the current study are available from the corresponding author on reasonable request.
